# Novel Role of Endothelial Derived Exosomal HSPA12B in Regulating Macrophage Inflammatory Responses in Polymicrobial Sepsis

**DOI:** 10.3389/fimmu.2020.00825

**Published:** 2020-05-07

**Authors:** Fei Tu, Xiaohui Wang, Xia Zhang, Tuanzhu Ha, Yana Wang, Min Fan, Kun Yang, P. Spencer Gill, Tammy R. Ozment, Yuan Dai, Li Liu, David L. Williams, Chuanfu Li

**Affiliations:** ^1^Department of Surgery, James H. Quillen College of Medicine, East Tennessee State University, Johnson City, TN, United States; ^2^The Center of Excellence in Inflammation, Infectious Disease and Immunity, James H. Quillen College of Medicine, East Tennessee State University, Johnson City, TN, United States; ^3^Department of Geriatrics, The First Affiliated Hospital of Nanjing Medical University, Nanjing, China

**Keywords:** sepsis, HSPA12B, macrophages, inflammatory responses, exosomes

## Abstract

Endothelial cell dysfunction contributes to sepsis induced initiate immune response and the infiltration of immune cells into organs, resulting in organ injury. Heat shock protein A12B (HSPA12B) is predominantly expressed in endothelial cells. The present study investigated whether endothelial HSPA12B could regulate macrophage pro-inflammatory response during sepsis. Wild type (WT) and endothelial cell-specific HSPA12B deficient (HSPA12B^–/–^) mice were subjected to CLP sepsis. Mortality and cardiac function were monitored. Higher mortality, worsened cardiac dysfunction, and greater infiltrated macrophages in the myocardium and spleen were observed in HSPA12B^–/–^ septic mice compared with the WT septic mice. The serum levels of TNF-α and IL-1β were higher and the levels of IL-10 were lower in HSPA12B^–/–^ septic mice than in WT septic mice. Importantly, endothelial exosomes contain HSPA12B which can be uptaken by macrophages. Interestingly, endothelial exosomal HSPA12B significantly increases IL-10 levels and decreases TNF-α and IL-1β production in LPS-stimulated macrophages. Mechanistic studies show that endothelial exosomal HSPA12B downregulates NF-κB activation and nuclear translocation in LPS stimulated macrophages. These data suggest that endothelial HSPA12B plays a novel role in the regulation of macrophage pro-inflammatory response via exosomes during sepsis and that sepsis induced cardiomyopathy and mortality are associated with endothelial cell deficiency of HSPA12B.

## Introduction

Sepsis is defined as life-threatening organ dysfunction caused by a dysregulated host innate and inflammatory response to infection (1). In the United States, nearly 270, 000 cases of sepsis occur yearly and the mortality of sepsis is from 30 to 50% (2). Cardiovascular dysfunction is a major complication associated with sepsis induced morbidity and mortality. Cardiac dysfunction in sepsis/septic shock is associated with high mortality rates as high as 70% (3).

Endothelial cell dysfunction contributes to sepsis induced mortality and organ dysfunction (4). Heat shock protein A12B (HSPA12B) is a member of HSP70 family and is predominately expressed in endothelial cells (5). Previous studies have shown that transgenic mice with endothelial cell specific overexpression of HSPA12B exhibits a protection against endotoxemia or myocardial ischemia induced cardiac dysfunction (6, 7). Increased expressing of HSPA12B in endothelial cells attenuates LPS-induced adhesion molecules expression and pro-inflammatory cytokine production via activation of the PI3K/Akt signaling (8). These data indicate that endothelial cell HSPA12B may be an endogenous mechanism that serves to protect the host during sepsis.

Macrophages play a critical role in mediating innate immune and inflammatory responses to sepsis challenge (9, 10). Increasing evidence demonstrates that macrophages play different roles during different stages of sepsis via the regulation of innate immune and inflammatory responses (10). In general, macrophage polarization has been divided into two groups, i.e., pro-inflammatory (M1) and anti-inflammatory (M2) phenotypes (11). In the early stage of sepsis, macrophages polarize toward the M1 phenotype, resulting in production of pro-inflammatory cytokines (11). In the late stage of sepsis, however, macrophages frequently exhibit an M2 phenotype that regulates anti-inflammatory mediators (12). However, these heterogeneous functions of macrophages are highly dependent on the microenvironment conditions (13). Importantly, recent evidence suggests that there is a crosstalk between endothelial cells and macrophages during sepsis (14). It would be interesting to investigate whether endothelial HSPA12B could regulate macrophage inflammatory responses during sepsis.

Exosomes have been demonstrated to play an important role in cell-cell communication (15). Exosomes are one subset of extracellular vehicles (EVs) that can be secreted from most cells (16). Recent studies have shown that exosomes are involved in the regulation of immune and inflammatory responses (17). Macrophage exosomes have been reported to alter endothelial cell migration and inflammation (18, 19). However, whether endothelial exosomes can regulate macrophage inflammatory responses during sepsis have not been investigated.

The present study showed that endothelial specific deficiency of HSPA12B (HSPA12B^–/–^) exhibited increased mortality and worsened cardiac dysfunction in a model of polymicrobial sepsis. We demonstrated that endothelial HSPA12B are released via exosome secretion during sepsis and that macrophages can uptake endothelial HSPA12B containing exosomes in both *in vivo* and *in vitro*. HSPA12B carried by exosomes attenuated macrophage inflammatory response to LPS stimulation via downregulation of NF-κB activation.

## Materials and Methods

### Animals

Endothelial specific HSPA12B knockout mice (HSPA12B^–/–^) were generated by cross-breeding the conditionally targeted HSPA12B mice with C57BL/6.Cg-Tg (Tek-cre) strain which carries Cre recombinase under the control of the Tek promoter. HSPA12B^–/–^ mice and age matched wild type (WT) C57BL/6 mice were used for experiments. The mice were maintained in the Division of Laboratory Animal Resources at East Tennessee State University. The experiments outlined in this manuscript conform to the Guide for the Care and Use of Laboratory Animals published by the National Institutes of Health (8th edition, 2011). The animal care and experimental protocols were approved by the Eastern Tennessee State University Committee on Animal Care.

### Murine Polymicrobial Sepsis Model

Murine polymicrobial sepsis was induced by cecal ligation and puncture as described previously (20–24). To induce of a less server polymicrobial sepsis, mice were anesthetized by 5.0% isoflurane. A midline incision was made on the anterior abdomen and the cecum was exposed and 1/3 of cecum was ligated with a 4-0 suture. One puncture was made at the end of cecum with a 23-gauge needle and feces were extruded from the hole. The abdomen was then closed in 2 layers. Sham surgically operated mice served as sham control. Immediately following surgery, a single dose of resuscitative fluid (lactated Ringer’s solution, 50 mL/kg body weight) was administered by subcutaneous injection (21, 23).

### Echocardiography

Transthoracic 2-dimensional M-mode echocardiogram and pulsed wave Doppler spectral tracings (Toshiba Aplio 80 Imaging System, Tochigi, Japan) were used to measure left ventricular (LV) wall thickness, LV end-systolic diameter, and LV end-diastolic diameter. Percentage of fractional shortening (% FS) and ejection fraction (EF %) were calculated as described previously (20, 21, 23).

### Measurement of Serum Levels of Lactate, Aspartate Aminotransferase, and Creatine Kinase

The serum levels of lactate were assessed with the Lactate Colorimetric Assay Kit (Millipore Sigma). Serum levels of aspartate aminotransferase (AST), and creatine kinase, were measured with commercially available kits according to the instructions provided by the manufacturer (Millipore Sigma).

### Tissue Accumulation of Macrophages

Accumulation of macrophages in heart tissues were examined using immunostaining with anti F4/80 antibody (Cell Signaling Technology). Three slides from each block were evaluated, counterstained with hematoxylin, and examined with confocal microscopy. The results are expressed as the numbers of immune cells/field (×40) (20, 21, 24).

### Immunofluorescent Staining

The procedure of the immunofluorescent staining was based on the protocol provided by Cell Signaling Technology. Briefly, cells were grown on the coverslips in a multiwell plate, fixed with 4% formaldehyde for 15 min in room temperature and washed for three times with PBS for 5 min each. Cells were permeabilized with ice-cold 100% methanol for 10 min at −20^*o*^C followed by rinsing in PBS for 5 min. The cells were incubated with 10% goat serum for 60 min at room temperature followed by incubation with the primary antibody that was diluted with 10% goat serum, at 4^*o*^C overnight according to the instruction provided by manufacturer. After washing for three times with PBS for 5 min each, the cells were incubated with a fluorochrome-conjugated secondary antibody for 60 min at room temperature in dark. The cells were washed with PBS for three times, covered by antifade reagent with DAPI and examined with confocal microscope.

### Transfection of Endothelial Cells With Adenovirus Expressing HSPA12B (Ad-HSPA2B)

Human umbilical vein endothelial cells (HUVECs) were cultured in vascular cell basal medium supplemented with endothelial cell growth kits (ATCC). After reaching 70–80% confluence, HUVECs were transfected with adenovirus expressing HSPA12B (Ad-HSPA12B, MIO = 10) or adenovirus expressing GFP. Six hours after transfection, the cells were washed and incubated with fresh medium overnight followed by LPS stimulation for 4 h. The cells were harvested and the cellular proteins were prepared for Western blot (8, 25).

### Transfection of Macrophages With Adenovirus Expressing HSPA12B (Ad-HSPA2B)

The procedure for transfection of macrophages with Ad-HSPA12B was according to K2 transfection system (Biontex). Briefly, when macrophages reached to 70%–80% confluence, the cells were pre-treated with the K2 multiplier for 2 h. K2 transfection reagent and Ad-HSPA12B or Ad-GFP were mixed at 1:2 ratio and incubated at room temperature for 30 min. The mixed transfection reagent was added into cultured macrophages. Six hours after transfection, the cells were washed and incubated with fresh culture medium overnight. The transfection efficiency was examined by viewing green fluorescence in the cells. And cells were ready for experiments.

### Isolation of Exosomes

Human umbilical vein endothelial cells were incubated with FBS-free medium. The medium were collected and centrifuged at 2000*g* for 30 min to remove debris. The supernatants were transferred into a new tube. Thirty percent (30%) PEG6000 (Sigma-Aldrich) reagent was mixed with the supernatant at ratio of 1:2 by votexing until there is a homogenous solution. The mixture was incubated at 2∼8^*o*^C overnight and centrifuged at 3000*g* for 30 min. The supernatant was removed. Exosomes in the pellet were resuspended in cold PBS and the exosome markers were examined by Western blot.

### Flow Cytometric Analysis

The procedure of flow cytometry was based on the protocol from BD Biosciences. Briefly, cells were harvested from blood and spleen tissues and prepared as single cell suspensions. Red blood cells were lysed using BD Pharmingen’s PharM Lyse^TM^ (Cat. No.555899) solution. The cell suspensions were incubated with Lyse^TM^ buffer at room temperature for 15 min in the dark and centrifuged at 200*g* for 5 min at 10^*o*^C. The supernatants were carefully removed. The pellets were suspended with 1x cold wash buffer (PBS/0.1% NaN_3_/1.0%FBS) followed by centrifugation at 350*g* for 5 min. Cells were counted, and reconstituted in staining buffer (BD Biosciences) to a concentration of 1 × 10^6^ cells/ml. Subsequently, cells will be incubated with LIVE/DEAD Fixable Dead Cell Stain single-color dyes (Invitrogen) for 30 min at room temperature. After one rinse with washing buffer, cells will be incubated with anti- Fc III/II (clone 2.4G2) antibody (BD Pharmingen) for 15 min and labeled at 4°C overnight followed by incubation with rat anti-mouse primary antibodies. Finally, cells will be washed twice, resuspended in stain buffer and immediately analyzed with a Becton Dickinson Fortessa X20 flow cytometer (BD Biosciences). Monocytes/macrophages were identified by CD11b^+^/F4/80^+^ cells and pro-inflammatory macrophages in spleen were identified by CD11b^+^/F4/80^+^/Ly6C^+^/CCR2^+^. Data analysis and quantification were performed using FlowJo. Antibody dilutions were adjusted for specific experiment according to manufacture manual.

### Western Blot

Western blot was performed as described previously (20, 21, 23, 24). In brief, cellular proteins were separated by sodium dodecyl sulfate–polyacrylamide gel electrophoresis and transferred onto Hybond enhanced chemiluminescence (ECL) membranes (Amersham Pharmacia, Piscataway, NJ, United States). The ECL membranes were incubated with the appropriate primary antibodies followed by peroxidase-conjugated secondary antibody, which was purchased from Cell Signaling Technology, Inc. The signals were quantified using the G: Box gel imaging system by Syngene (Frederick, MD, United States).

### Cytokine Assay

The levels of TNF-α, IL-1β, and IL-10 in the serum were measured by enzyme-linked immunosorbent assay (ELISA) using OptEIA cytokine kits (Pepro Tech) as described previously (20, 21, 24, 26).

### Statistical Analysis

The data are expressed as mean ± standard error. Comparisons of data between groups were made using one-way analysis of variance (ANOVA), and Tukey procedure for multiple-range tests was performed. The log-rank test was used to compare group survival trends. Probability levels of ≤0.05 were used to indicate statistical significance.

## Results

### Increased Mortality and Worsened Cardiac Dysfunction in HSPA12B^–/–^ Septic Mice

**FIGURE 1:**
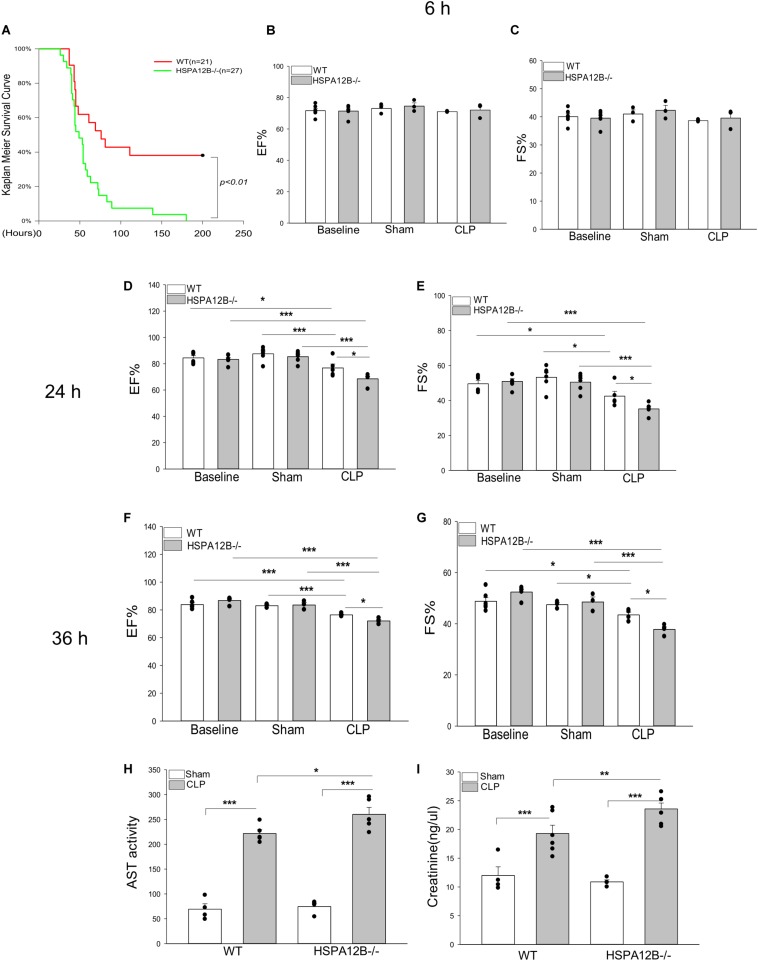
Increased mortality and severer cardiac dysfunction in HSPA12B^–/–^ septic mice. **(A)** WT and HSPA12B^–/–^ mice were subjected to a CLP sepsis. The survival outcome was monitored for up to 200 h following induction of CLP (*n* = 13∼14/group). **(B–G)** Cardiac function was examined by echocardiography at 6, 24, and 36 h after CLP. EF%, ejection fraction. %FS, fractional shortening. Serum levels of aspartate aminotransferase (AST; **H**), creatinine **(I)** were measured by commercially available ELISA kits. *n* = 3∼6/group. *P < 0.05, **P < 0.01, and ***P < 0.001 compared with indicated groups.

To investigate the effect of endothelial HSPA12B on the survival outcome and cardiac function during sepsis, wild type (WT) and HSPA12B^–/–^ mice were subjected to cecal ligation and puncture (CLP) induced sepsis and survival outcome and cardiac function were monitored. As shown in Figure 1A, HSPA12B^–/–^ septic mice exhibited an exacerbation of mortality compared with WT septic mice. The time to 50% mortality of WT septic mice was at ∼75 h, 62% mortality was at ∼110 h, and maintained duration of the observation periods of time (200 h). In contrast, the time to 100% mortality of HSPA12B^–/–^ septic mice was 63 h. The data suggests that endothelial HSPA12B plays a role in limiting mortality in CLP sepsis.

Sepsis induced cardiomyopathy contributes to mortality and mobility of sepsis (27, 28). Figures 1B–G show that cardiac function was not reduced at 6 h after induction of CLP sepsis. However, ejection fraction (EF%) and fractional shortening (FS%) were markedly reduced in both WT and HSPA12B^–/–^ septic mice 24 and 36 h after CLP sepsis, when compared with their respective sham controls. Importantly, the values of EF% and FS% in HSPA12B^–/–^ septic mice were markedly lower (12.1% and 19% at 24 h, 6.1% and 15.1% at 36 h) than in WT septic mice. In addition, the serum levels of AST and creatinine in the HSPA12B^–/–^ septic mice were significantly greater by 17.5 and 22.2% than in WT septic mice (Figures 1H,I). The data suggests that endothelial HSPA12B could play an important role in the protection against sepsis induced organ injury.

### Increased Accumulation of Macrophages in the Myocardium of HSPA12B^–/–^ Septic Mice

**FIGURE 2:**
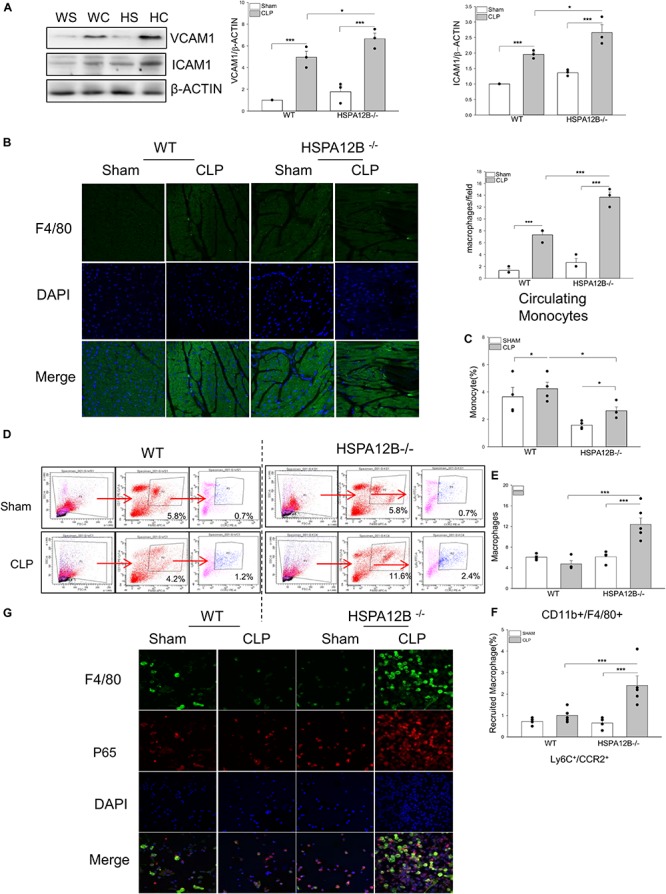
Increased macrophage populations in the myocardium, circulation and spleen in HSPA12B^–/–^ septic mice. WT and HSPA12B^–/–^ mice were subjected to a CLP sepsis. Blood, spleen, and heart tissues were harvested at 36 h after CLP for the examination of macrophage populations. **(A)** Western blot analysis of myocardial vascular cell adhesion molecule-1 (VCAM-1) and intercellular adhesion molecule (ICAM-1). *n* = 3/group. **(B)** Heart tissues were sectioned and stained with anti-F4/80 antibody (green). Nucleus were stained with DAPI (blue). *n* = 3/group. **(C)** Flow cytometry analysis of the monocytes in blood **(C)** and the macrophages in spleen **(D)** with anti-CD11b^+^F4/80^+^ antibodies **(E)** and the pro-inflammatory macrophages in spleen with anti-Ly6C^+^/CCR2^+^ antibodies from CD11b^+^F4/80^+^ macrophages **(F)** *n* = 3∼5/group. **(G)** Immunofluorescent staining of peritoneal macrophages from septic mice, with anti-F4/80 antibody (green), and anti-p65 antibody (red). Nucleus were stained with DAPI (blue). WS, WT sham; WC, WT CLP; HS, HSPA12B^–/–^ sham; HC, HSPA12B^–/–^ CLP. *P < 0.05 and ***P < 0.001 compared with indicated groups.

Increased accumulation of immune cells in the myocardium contributes to septic cardiomyopathy (29, 30), while increased expression of adhesion molecules facilitate immune cell infiltration into in the heart tissues (31). As shown in Figure 2A, the levels of VCAM-1 and ICAM-1 in the myocardium of HSPA12B^–/–^ septic mice were significantly greater than in WT septic mice. Figure 2B shows that the numbers of F4/80^+^ macrophages in the myocardium of HSPA12B^–/–^ septic mice were significantly greater than in WT septic mice. The data indicates that endothelial cell HSPA12B could play a role in the attenuation of macrophage infiltration into the myocardium through suppressing myocardial adhesion molecule expression during sepsis.

### Increased Monocyte/Macrophage Populations in the Circulation and the Spleen in HSPA12B^–/–^ Septic Mice

Activation of monocytes/macrophages has been demonstrated to play a critical role in sepsis induced organ injury (32, 33). We examined whether endothelial HSPA12B could regulate monocyte/macrophage populations in spleen and circulation after induction of CLP sepsis. Figure 2C shows that the numbers of circulation monocytes were markedly decreased in HSPA12B^–/–^ septic mice, but not in WT septic mice. However, the numbers of splenic macrophages in HSPA12B^–/–^ septic mice were greater than in WT septic mice (Figures 2D,E). Flow cytometry analysis shows that there are more Ly6C^+^/CCR2^+^ cell populations in the spleen from HSPA12B^–/–^ septic mice, when compared with WT septic mice (Figures 2D,F). To investigate whether NF-κB is activated in septic macrophages, we isolated peritoneal macrophages from WT and HSP12B^–/–^ mice and examined NF-κB subunit p65 nuclear translocation with immunostaining. As shown in Figure 2G, macrophages that were isolated from the peritoneal cavity of HSPA12B^–/–^ septic mice exhibit greater NF-κB p65 subunit nuclear translocation than in WT septic mice. These data indicate that endothelial HSPA12B may regulate macrophage response to CLP sepsis by attenuating NF-κB activation.

### High Levels of Serum Lactate and Inflammatory Cytokines in HSPA12B^–/–^ Septic Mice

**FIGURE 3:**
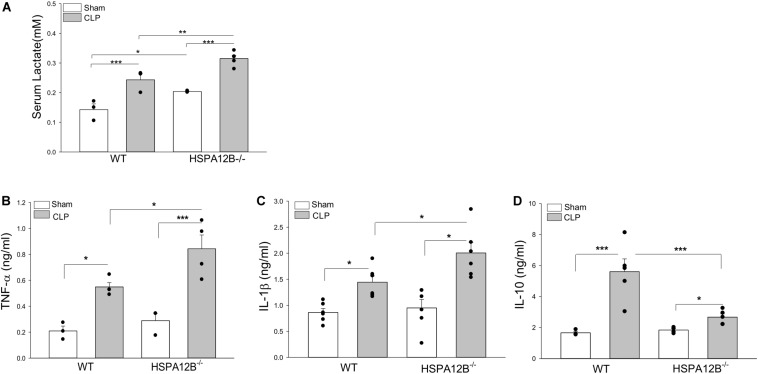
Increased serum levels of lactate and inflammatory cytokines in HSPA12B^–/–^ septic mice. WT and HSPA12B^–/–^ mice were subjected to a CLP sepsis. Serum was collected at 36 h after CLP sepsis. **(A)** Serum lactate levels at 36 h. **(B–D)** Serum levels of TNF-α **(B)**, IL-1β **(C),** and IL-10 **(D)** at 36 h after CLP. *n* = 3∼6/group *P < 0.05, **P < 0.01, and ***P < 0.001 compared with indicated groups.

Serum lactate levels serve as a biomarker for severity and mortality of sepsis (34). Figure 3A shows that CLP sepsis significantly increased serum lactate levels in both WT and HSPA12B^–/–^ mice compared with sham controls. However, the levels of serum lactate in HSPA12B^–/–^ septic mice were increased by 27.5% compared to WT septic mice. CLP sepsis also markedly increased the levels of serum TNF-α, IL-1β, and IL-10 in both WT and HSPA12B^–/–^ mice 24 h (data not shown) and 36 h after induction of CLP sepsis (Figures 3B–D). However, the serum TNF-α and IL-1 β levels in HSPA12B^–/–^ septic mice were markedly increased by 53.5 and 38.9% while IL-10 decreased by 52.7%, when compared with WT septic mice. These data indicate that endothelial HSPA12B could participate in the regulation of inflammatory cytokine production during CLP sepsis.

### Tissue Exosomes Isolated From WT Mice Contained Endothelial HSPA12B

**FIGURE 4:**
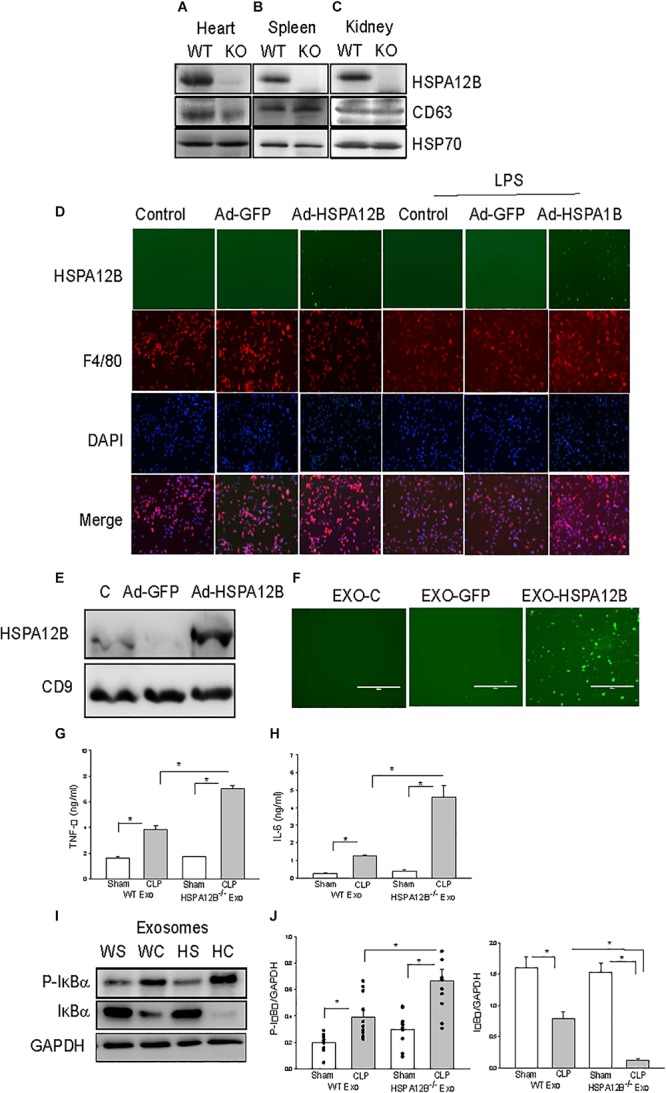
Macrophages uptake endothelial exosomes that contain HSPA12B. **(A–C)** Exosomes were isolated from heart **(A)**, spleen **(B)**, and kidney **(C)** of WT and HSPA12B^–/–^ mice. **(D)** Macrophages uptake endothelial HSPA12B from endothelial cell conditioned medium. HUVECs were transfected with Ad-HSPA12B-GFP or Ad-GFP. Twenty-four hours after transfection, the cultured medium were collected as the endothelial conditioned medium (ECM). Macrophages (Raw 264.7) were incubated with ECM. Endothelial HSPA12B in macrophages were examined with fluorescent microscope. HSPA12B exhibits green. Macrophages were strained with anti-F4/80 antibody. Nucleus were stained with DAPI. **(E)** Western blot analysis of exosomal HSPA12B levels, CD9 as a loading control. **(F)** Exosomes were isolated from ECM. Macrophages (Raw 264.7) uptake endothelial exosomes that contain high levels of HSPA12B. Macrophages were incubated with endothelial exosomes that were isolated from ECM for 1 h. Endothelial HSPA12B (green) in macrophages was viewed with fluorescent microscope. Serum exosomes were isolated from WT and HSPA12B^–/–^ sham and septic mice. Macrophages were treated with exosomes for 12 h. The medium was harvested for analysis of TNFα **(G)** and IL-6 **(H)** production by ELISA kits (*n* = 4–6/group). **(I)** Murine macrophages (Raw 264.7) were treated with the isolated exosomes for 45 min. The cells were harvested for analysis of IκBα phosphorylation **(J)** by Western blot (*n* = 4–6/group). C, control; L, LPS; WS, WT sham; WC, WT CLP; HS, HSPA12B^–/–^ sham; HC, HSPA12B^–/–^ CLP; Exo, exosomes. *P < 0.05 compared with indicated groups.

Exosomes have been demonstrated to play a critical role in mediating cell-cell and tissue-tissue communication (35, 36). We investigated whether endothelial HSPA12B could regulate macrophage activation and inflammatory cytokine production via exosome secretion. Exosomes were isolated from tissues and HSPA12B levels in the isolated exosomes were examined by Western blot. Figures 4A–C shows that the exosomes isolated from the heart, kidney, and spleen tissues from WT mice contain HSPA12B, but it is not present in HSPA12B^–/–^ mice. Since HSPA12B is mainly expressed in endothelial cells (37), the data indicates HSPA12B could be released from endothelial cells via exosome secretion.

### Endothelial HSPA12B Could Be Uptaken by Macrophages

To define whether HSPA12B containing exosomes can be uptaken by macrophages, we transfected endothelial cells (HUVECs) with adenovirus expressing HSPA12B (Ad-HSPA12B) or Ad-GFP which served as vector control. Twenty-four hours after transfection, we harvested the medium as an endothelial conditioned medium (ECM), incubated the macrophages (Raw 246.7) with the ECM, and examined whether endothelial HSPA12B could be uptaken by macrophages. As shown in Figure 4D, green fluorescence that indicates endothelial HSPA12B appeared in the macrophages that were incubated with ECM from the HUVECs transfected with Ad-HSPA12B (containing green fluorescence), but not from Ad-GFP transfected HUVECs. Interestingly, stimulation of HUVECs with LPS increased HSPA12B in the ECM as evidenced showing that macrophages contained more fluorescence than control group. The data indicates that LPS stimulation could promote endothelial cells to release HSPA12B via exosome secretion. To confirm our finding, we transfected HUVECs with Ad-HSPA12B or Ad-GFP for 24 h, isolated exosomes from cultured medium, and incubated macrophages with the isolated exosomes. Figures 4E,F shows that endothelial HSPA12B containing exosomes were uptaken by macrophages. The data demonstrated that endothelial cell HSPA12B could be transmitted into macrophages via uptaking exosomes.

### HSPA12B^–/–^ Septic Exosomes Increased Inflammatory Cytokine Production and NF-κB Activation in Macrophages

We observed increased pro-inflammatory cytokine levels in HSPA12B^–/–^ septic mice (Figure 3). We examined whether septic exosomes would play a role in the increased pro-inflammatory cytokine production in macrophages. We isolated serum exosomes from WT and HSPA12B^–/–^ septic mice and treated macrophages with the isolated serum exosomes. Figures 4G,H show that incubation of macrophages with WT septic exosomes significantly increased TNFα (133.9%) and IL-6 (427.9%) production, when compared with the WT sham exosome treated group. However, incubation of macrophages with HSPA12B^–/–^ septic exosomes markedly increased greater production of TNFα (184%) and IL-6 (360%) compared with WT septic exosome treated group. Activation of NF-κB regulates inflammatory cytokine production (38). IκBα phosphorylation and degradation are crucial steps for NF-κB translocation and binding activity (39). We examined the effect of septic exosomes on NF-κB activation in macrophages. Figures 4I,J shows that WT septic exosome treatment markedly increased the levels of phosphorylated IκBα (154.1%) and decreased total IκBα levels (102.3%) compared with the WT sham exosome treated group. Interestingly, HSPA12B^–/–^ septic exosome further increased the level of phosphorylated IκBα by 89.3% and decreased total IκBα levels by 90.2%, when compared with WT septic exosome treated group. The data suggests that septic exosomes could induce pro-inflammatory cytokine production and NF-κB activation in macrophages. Septic exosomes that were deficiency of HSPA12B exhibited a greater effect than in WT exosomes on NF-κB activation mediated pro-inflammatory cytokine production in macrophages.

### Endothelial Conditioned Medium (ECM) Attenuated LPS-Stimulated Inflammatory Cytokine Production in Macrophage

**FIGURE 5:**
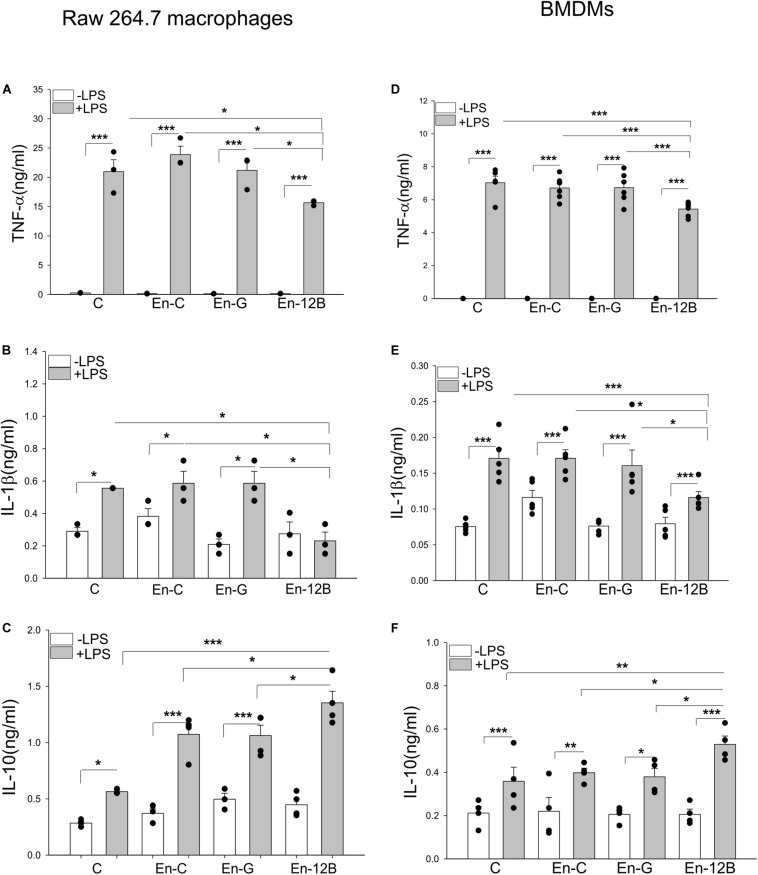
Endothelial conditioned medium (ECM) altered macrophage response to LPS stimulation. Macrophages (Raw 264.7) were incubated with ECM followed by LPS (1 μg/ml) stimulation for 24 h. The medium were collected for the measurement of TNFα **(A)**, IL-1β **(B),** and IL-10 **(C)** by ELISA. **(D–F)** Bone marrow derived macrophages (BMDM) were incubated with ECM followed by LPS (1 μg/ml) stimulation for 24 h. The medium were collected for the measurement of TNF-α **(D)**, IL-1β **(E)** and increased IL-10 **(F)** by ELISA. C, control; En-C, endothelial medium control; En-G, Ad-GFP endothelial medium; En-12B, Ad-HSPA12B endothelial medium control. *n* = 3∼6/group. *P < 0.05, **P < 0.01, and ***P < 0.001 compared with indicated groups.

To investigate that HSPA12B released from endothelial cells could regulate the inflammatory cytokine production in macrophages, we first incubated macrophages with ECM before the cells were stimulated with LPS. Figures 5A–C show that LPS stimulation markedly increased the levels of TNFα (A), IL-1β (B) and IL-10 (C) compared with unstimulated control. However, ECM harvested from Ad-HSPA12B transfected HUVECs, but not from Ad-GFP transfected HUVECs significantly attenuated LPS stimulated production of TNFα by 20.4% and IL-1β by 60.6% in macrophages. Interestingly, ECM harvested from Ad-HSPA12B transfected endothelial cells further enhanced LPS stimulated IL-10 production (by 27.3%) in macrophages. Similarly, incubation of bone marrow derived macrophages (BMDMs) with ECM collected from Ad-HSPA12B transfected HUVECs markedly suppressed LPS stimulated TNFα and IL-1β production and enhanced IL-10 production (Figures 5D–F). The data suggests that endothelial HSPA12B could regulate macrophage response to LPS stimulation.

### Endothelial HSPA12B-Containing Exosomes Regulated Inflammatory Responses in Macrophages

**FIGURE 6:**
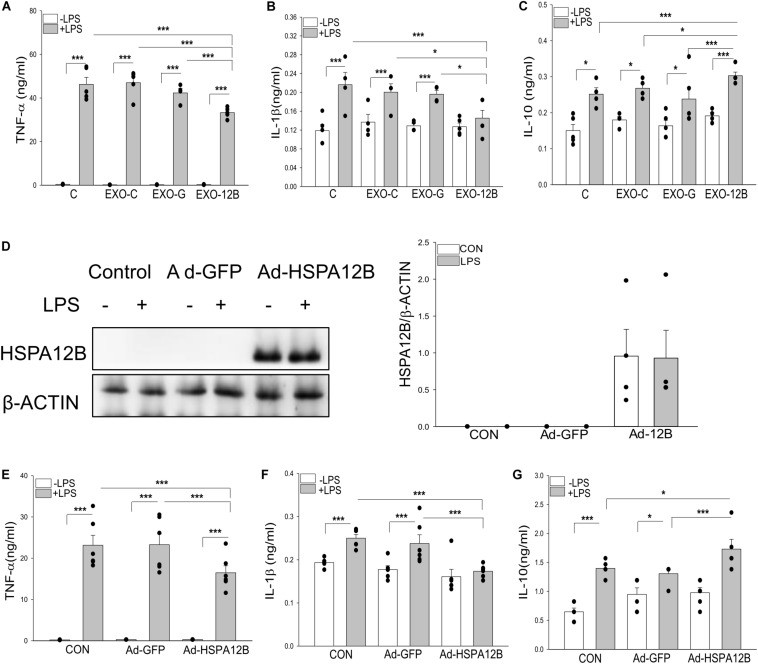
Endothelial exosomes altered macrophage response to LPS stimulation. Exosomes were isolated from HEVECs that were transfected with Ad-HSPA12B or Ad-GFP followed by LPS stimulation for 24 h. Macrophages (RAW 264.7) were incubated with the isolated exosomes before LPS (1 μg/ml) stimulation for 24 h. The medium were collected for measurement of TNF-α **(A)**, IL-1β **(B),** and IL-10 **(C)** with ELISA. *n* = 4 independent experiments/group. **(D)** Macrophages (Raw264.7) were transfected with Ad-HSPA12B or Ad-GFP for 6 h by K2 transfection system then changed with fresh medium overnight before the cells were stimulated with LPS (1 μg/ml) for 1 h. **(D)** Western blot analysis of HSPA12B levels in macrophages. The **(E,F)** Macrophages (Raw 264.7) were transfected with Ad-HSPA12B or Ad-GFP for 6 h by K2 transfection system then changed with fresh medium overnight before the cells were stimulated with LPS (1 μg/ml) for 24 h. Cultured medium was collected for the measurement of TNF-α **(E)**, IL-1β **(F),** and increased IL-10 **(G)** with ELISA. *n* = 3∼5 independent experiments/group. *P < 0.05 and ***P < 0.001 compared with indicated groups.

We then investigated whether exosomal HSPA12B released by endothelial cells could be responsible for ECM regulated macrophage response to LPS stimulation. Endothelial exosomes were isolated from ECM that was collected from cultured endothelial cells (HUVECs) transfected with or without Ad-HSPA12B or Ad-GFP (defined as exosomes/control, exosomes/GFP and exosomes/HSPA12B). Macrophages were incubated with the isolated endothelial exosomes followed by LPS stimulation. Figures 6A–C show that LPS stimulation markedly increased TNFα (A), IL-1β (B), and IL-10 (C) production in macrophages. Importantly, exosomes/HSPA12B, but not exosomes/GFP or exosomes/control significantly attenuated LPS induced increases in TNFα and IL-1β levels and enhanced LPS stimulated IL-10 production in macrophages. The data suggests that exosomes that contain high levels of HSPA12B could attenuate the response of macrophages to LPS challenge.

To confirm the role of HSPA12B in exosomes/HSPA12B regulating macrophage response to LPS stimulation, we directly transfected macrophages with Ad-HSPA12B or Ad-GFP 24 h before the cells were stimulated with LPS. Figure 6D shows that high levels of HSPA12B were observed in the macrophages that were directly transfected with Ad-HSPA12B. Figures 6E–G show that transfection of Ad-HSPA12B, but not Ad-GFP attenuated LPS stimulated TNFα and IL-1β production and enhanced LPS stimulated IL-10 in macrophages. The data demonstrated that endothelial HSPA12B regulates the response of macrophages to LPS stimulation.

### Endothelial HSPA12B-Containing Exosomes Downregulated NF-κB Activation in LPS Stimulated Macrophages

**FIGURE 7:**
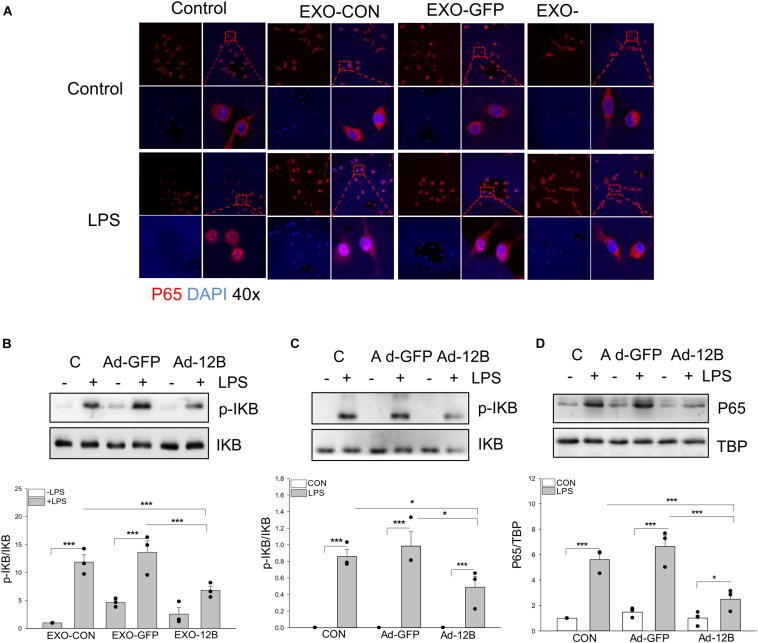
Endothelial exosomes that contain high levels of HSPA12B altered NF-κB activation in LPS stimulated macrophages. Exosomes were isolated from HEVECs that were transfected with Ad-HSPA12B or Ad-GFP followed by LPS stimulation. Macrophages (RAW 264.7) were incubated with the isolated exosomes for 2 h prior to LPS (1 μg/ml) stimulation for 1 h. **(A)** Immunostaining of NF-κB subunit p65 (red) nuclear translocation. The nucleus was stained with DAPI (blue). **(B)** Macrophages (RAW 264.7) were incubated with the endothelial exosomes (5 μg/ml) for 8 h followed by LPS stimulation for 1 h. Phosphorylated IκBα and total IκBα were assessed with Western blot. **(C,D)** Macrophages (Raw 264.7) were transfected with Ad-HSPA12B or Ad-GFP for 6 h by K2 transfection system then changed with fresh medium overnight before the cells were stimulated with LPS (1 μg/ml) for 1 h. Western blot analysis of the levels of phosphorylated IκBα and total IκBα in the cytoplasm and NF-κB subunit p65 levels in the nucleus. *N* = 3∼4/group. *P < 0.05 and ***P < 0.001 compared with indicated groups.

To investigate the mechanisms by which endothelial HSPA12B regulates macrophage response to LPS stimulation, we examined NF-κB activation in macrophages. Confocal microscopy examination shows that LPS stimulation induced NF-κB subunit p65 nuclear translocation (Figure 7A). Western blot analysis shows that LPS stimulation increased the levels of phosphorylated IκBα (Figure 7B) in macrophages. However, exosomes/HSPA12B administration markedly attenuated LPS induced NF-κB subunit p65 nuclear translocation (Figure 7A), IκBα phosphorylation (Figure 7B). In addition, directly transfection of macrophages with ad-HSPA12B-GFP, but not ad-GFP significantly attenuated LPS induced IκBα phosphorylation (Figure 7C) and NF-κB subunit p65 nuclear translocation (Figure 7D). These data suggest that endothelial HSPA12B could directly regulates macrophage response to LPS stimulation via downregulation of NF-κB activation.

## Discussion

The present study demonstrates that endothelial HSPA12B exerts a protective effect on sepsis-induced mortality and cardiomyopathy. The mechanisms involve release of endothelial HSPA12B via secretion of exosomes, which are subsequently uptaken by macrophages. Importantly, endothelial exosomal HSPA12B downregulates macrophage pro-inflammatory responses in sepsis. Exosomes have been demonstrated to play a critical role in mediating cell-to-cell and organ-to-organ communication (35, 36). Our data provides compelling evidence that endothelial cells can regulate macrophage pro-inflammatory function during sepsis through exosomal HSPA12B.

Endothelial HSPA12B has been reported to attenuate endotoxemia (6) and cardiac injury due to myocardial infarction (7). The mechanisms involve activation of PI3K/Akt signaling and suppression of NF-κB activation (6, 8). Endothelial HSPA12B also protects vascular endothelial cells from sepsis induced acute lung injury (40). In the present study, we found that deficiency of HSPA12B in endothelial cells results in more severe cardiac dysfunction, decreased survival outcome, and increased pro-inflammatory cytokine production in polymicrobial sepsis. These findings suggest that endothelial HSPA12B plays an important role in limiting pro-inflammatory responses and protecting the host against sepsis induced cardiomyopathy and mortality.

To elucidate the mechanisms by which endothelial HSPA12B attenuates sepsis induced cardiomyopathy and organ injury, we focused on the role of endothelial HSPA12B in the regulation of macrophage inflammatory response to sepsis. Recent evidence highlighted the role of macrophage function in sepsis induced organ dysfunction (21, 29). We observed that endothelial HSPA12B deficiency results increased macrophage infiltration into the heart during CLP sepsis. Interestingly, there are no changes in the numbers of circulation and splenic monocytes/macrophages in HSPA12B^–/–^ sham mice, when compared with WT sham control. However, the numbers of circulation monocytes marked with F4/80^+^/CD11b^+^ in HSPA12B^–/–^ septic mice were markedly lower than in WT septic mice, while splenic Ly6C^+^/CCR2^+^ cell population in HSPA12B^–/–^ septic mice were greater than in WT septic mice. The data indicates that endothelial HSPA12B plays an important role in the regulation of monocyte/macrophage infiltration into the tissues. Although we do not fully understand the mechanisms by which endothelial HSPA12B regulates the infiltration of monocytes/macrophages into the tissues during polymicrobial sepsis at present, we observed that the levels of adhesion molecules in the myocardium were markedly increased in HSPA12B^–/–^ septic mice compared with WT septic mice. Increased adhesion molecule expression in HSPA12B^–/–^ septic mice could be responsible for increased infiltration of monocytes/macrophages into the tissues.

In addition, we observed that pro-inflammatory cytokine levels in HSPA12B^–/–^ septic mice were markedly greater than in WT septic mice, suggesting that endothelial HSPA12B also plays a role in regulating macrophage pro-inflammatory responses during sepsis. Previous studies have shown that there is crosstalk between endothelial cells and macrophages during sepsis and/or inflammatory diseases (41, 42). Endothelial cells and macrophages have been reported to engage via specific interactions to regulate vascular function (43, 44). Adult endothelial cells provide critical signals for the selective growth and differentiation of macrophages from several hematopoietic progenitors (45, 46). Thus, endothelial cells may serve as endogenous regulators of macrophage activation or suppression and by extension mediate innate immune and inflammatory responses. Indeed, we observed that the endothelial cell conditioned medium (ECM) generated from Ad-HSPA12B transfected endothelial cells markedly downregulated LPS induced inflammatory cytokine production by macrophages.

To elucidate the mechanisms by which endothelial HSPA12B regulates macrophage pro-inflammatory response during polymicrobial sepsis, we examined whether HSPA12B is released through exosome secretion. Interestingly, we found that endothelial cells release HSPA12B in exosomes and that exosomal HSPA12B is uptaken by macrophages. Exosomes have been demonstrated to play a critical role in cellular communication in both physiological and pathological conditions (35, 36). Exosomes contain a variety of bioactive molecules, including proteins, lipids, mRNA, and miRNAs from parent cells, which can be taken up by and affect the functions of recipient cells (5). Njock et al. reported endothelial vesicles (EVs) loaded with miRNAs suppress inflammatory response in monocytes/macrophages (41). He et al. reported the endothelial EVs modulated macrophage polarization toward M2 anti-inflammatory phenotype (47). Interestingly, we observed that exosomes isolated from tissues contain HSPA12B. *In vitro* data shows that HSPA12B can be transmitted from endothelial cells to macrophages via exosomes. Importantly, endothelial derived exosomal HSPA12B markedly downregulates LPS induced macrophage pro-inflammatory cytokine production with a concomitant upregulation of IL-10. To confirm our observation, we performed direct transfection of macrophages with Ad-HSPA12B and observed that transfected Ad-HSPA12B suppressed LPS induced pro-inflammatory cytokines and enhanced anti-inflammatory cytokine production. This data strongly supports the concept that endothelial derived exosomal HSPA12B can regulate macrophage response to pro-inflammatory stimuli. Our findings suggest that endothelial cell HSPA12B modulates macrophage response to sepsis via exosome secretion which mediates a crosstalk between endothelial cells and macrophages. Of greater importance, endothelial HSPA12B exerts an anti-inflammatory effect on macrophages in response to sepsis.

Activation of the NF-κB signaling pathway contributes to sepsis induced mortality and organ dysfunction (48). Our previous studies have demonstrated that targeting Toll-like receptor (TLR) mediated NF-κB activation increases survival outcome in ploymicriobial sepsis (21, 22). To elucidate the mechanisms of endothelial derived exosomal HSPA12B on macrophage pro-inflammatory responses, we examined the effect of endothelial derived exosomal HSPA12B on macrophage NF-κB activation and demonstrated that exosomal HSPA12B significantly suppresses NF-κB P65 subunit nuclear translocation in LPS stimulated macrophages. Our data suggests that endothelial derived exosomal HSPA12B regulates NF-κB activation in macrophages. Future studies are needed to elucidate the specific mechanisms by which exosomal HSPA12B suppresses NF-κB activation in LPS challenged macrophages.

To the best of our knowledge this is the first report demonstrating that endothelial cell HSPA12B can be released through exosome secretion and that exosomal HSPA12B mediates crosstalk between endothelial cells and macrophages during polymicrobial sepsis. The endothelial exosomal HSPA12B regulates macrophage pro-inflammatory cytokine production via attenuation of NF-κB activation.

## Data Availability Statement

The raw data supporting the conclusions of this article will be made available by the authors, without undue reservation.

## Ethics Statement

The animal care and experimental protocols were approved by the East Tennessee State University Committee on Animal Care.

## Author Contributions

FT and XW conceived of the presented idea. FT wrote the manuscript in consulation with from DW and CL. FT and XZ carried out the experiments, and support in experiments by TH, PG, and TO. TH, YW, MF, KY, YD, and LL contributed to the design of the research. DW and CL supervised the project.

## Conflict of Interest

The authors declare that the research was conducted in the absence of any commercial or financial relationships that could be construed as a potential conflict of interest.
